# The Place for Bookworms

**Published:** 1874-11

**Authors:** 


The Place for Bookworms.—We are never happier than when we can steal an hour from our duties and drop down in some quiet corner of a cosy book store to indulge in the supreme enjoyment of glancing through the pages of all the latest works of interest. No spot pleases us better for this purpose than the pleasant store of James Miller & Co., 647 Broadway. Here we find all that is entertaining and useful, besides a thousand works of art and interest not strictly in the line of literature. Mr. Knox, the manager of the house, picks out all the most attractive books and pictures, and invites us to fill to the full, of genuine delight. It is hard to pass without calling, and harder to depart after getting inside.

